# Emotional Effects of the Duration, Efficiency, and Subjective Quality of Sleep in Healthcare Personnel

**DOI:** 10.3390/ijerph16193512

**Published:** 2019-09-20

**Authors:** María del Carmen Pérez-Fuentes, María del Mar Molero Jurado, María del Mar Simón Márquez, Ana Belén Barragán Martín, José Jesús Gázquez Linares

**Affiliations:** 1 Department of Psychology, Faculty of Psychology, University of Almería, 04120 Almería, Spain; mpf421@ual.es (M.d.C.P.-F.); mmj130@ual.es (M.d.M.M.J.); msm112@ual.es (M.d.M.S.M.); abm410@ual.es (A.B.B.M.); 2 Department of Psychology, Faculty of Psychology, Universidad Politécnica y Artística del Paraguay, Asunción 1628, Paraguay; 3 Department of Psychology, Faculty of Psychology, Universidad Autónoma de Chile, 4780000 Santiago, Chile

**Keywords:** emotional effects, duration, efficiency, subjective quality, sleep

## Abstract

Considering that both sleep quality and duration are linked to psychological variables, this study analyzed the relationships between sleep components and emotional intelligence and the effects that sleep duration has on stress management and mood in a sample of nurses. The sample was made up of 1073 professionals. Data were collected by the Pittsburgh Sleep Quality Index and the Brief Emotional Intelligence Inventory for Senior Citizens. The results showed that the components of sleep quality were negatively related to stress management and mood. Furthermore, nurses who had short sleep patterns also had low moods and high stress levels. This study emphasizes the importance of subjective sleep quality as a necessary resource for professionals to manage stressful situations and mood and improve their relations with their patients and with each other.

## 1. Introduction

Sleep is one of the elements that helps with the achievement of physical and psychological wellbeing and is furthermore fundamental to emotional and cognitive development [1]. Thus, the effects of low-quality sleep influence a person’s individual and social development and functioning [2]. Because of this, and because it leads to a series of positive effects for individual health and wellbeing, sleep quality is generating strong interest in research. When discussing sleep quality, we refer to the subjective perception of sleep, considering a set of quantitative components, such as sleep duration, latency, and efficiency, and qualitative components, such as subjective sleep quality, disturbances, daytime dysfunction, and use of medication, which are the more subjective aspects of sleep [3]. Sleep disorders, poor quality sleep, and short duration of sleep are related negatively to wellbeing [4,5] and quality of life related to health [6,7], with harmful consequences and effects on health itself.

In this way, adequate and good-quality sleep is vitally important for health. Recently, prolonged use of digital media near bedtime has shown negative effects on sleep, such as reduced rest time and a late bedtime [8]. Furthermore, during the night, more and more people are leaving their mobile phone on, causing a greater number of sleep interruptions which are associated with fatigue and decreased mood [9]. In addition, more and more users are present on social networks and digital media; dependence on these networks can cause insomnia [10] and increase the presence of daily cognitive failures, this effect being mediated by the decrease in sleep quality [11]. In this way, the excessive use of digital media, linked to new social demands and socio-economic advances, affects the well-being, quality of life, and sleep patterns of workers [12].

The prevalence of sleep-related problems is higher in social service professions, such as healthcare [13]. Studies [14,15] have shown that lack of sleep is a common problem among nurses and that the duration of sleep varies with age [16]. In recent years, the prevalence of insomnia, which is the most important sleep-related disorder [17], and other sleep complaints have increased among healthcare personnel thus affecting the subjective quality of sleep [18]. Some studies have mentioned that the subjective quality of sleep is not related to working in shifts [19,20]. However, other studies have demonstrated a relationship between shifts and poor sleep quality in nurses [21,22]. Moreover, the study by Surani, Murphy, and Shah [19] showed that sleep privation has negative effects on nurses’ performance, causing them to make mistakes and even have accidents. People who work on different shifts also suffer from health disorders [23], for example, in eating patterns [24], which in turn are closely related to sleep quality and self-esteem [25]. At present, poor sleep quality among healthcare professionals is not only a risk factor for performing their services, but a critical problem in the healthcare system [26], which can lead to development of mood disorders and deficient stress management. Thus, some studies have found that the duration and quality of sleep and emotion management by healthcare professionals are closely connected [27,28]. This study therefore examined the relationship between sleep quality and its emotional effects on nursing personnel.

### 1.1. Sleep Quality and Emotion Management

Both sleep quality and duration are associated with psychological variables affecting the quality of life, including emotion management. Healthcare workers struggle daily with a diversity of situations in which emotions have a fundamental role, so cognitive emotion regulation [29,30] is especially important in the performance of those professions [31]. In addition, sleep regulation and emotions interact in anxiety and depression disorders [32]. Research in neuroscience has found that continued sleep deficiency impairs subjective mood, through altered tonsil activity and its functional connectivity [33]. Therefore, emotional regulation is strongly linked to circadian rhythms, which relates the affective clinical symptomatology with sleep disturbances [34]. However, in the study by Denis et al. [35], the presence of emotionally insensitive traits (such as lack of empathy, indifference to the feelings of others, or lack of emotional reactivity) did not show to be associated with lack of sleep.

In this way, emotional intelligence, considered as the ability to recognize and manage experiences and responses in oneself and others, specified their integration to guide the actions and thoughts achieved [36], is closely related to behaviors and employee satisfaction in jobs with high emotional loads [37]. Among the components of emotional intelligence are intrapersonal skills (which include the individual’s emotional self-awareness and assertiveness), interpersonal skills (which refers to social awareness), stress management (which includes management and regulation emotional), adaptability (in reference to flexibility and problem solving), and mood (which refers to the state of optimism and motivation of the individual) [38].

Therefore, emotional intelligence components, such as stress management and mood, have a protective effect against burnout [39,40] and engagement [41] in both nursing professionals and Health Science students [42,43]. Similarly, emotional intelligence, and specifically, stress management and mood, are closely related to sleep quality. Previous studies have emphasized the effect of sleep duration on both components in nursing professionals [44,45]. Lemola, Ledermann, and Friedman [46] analyzed the relationship between subjective wellbeing and sleep duration and sleep quality, and found a relationship between them mediated by the effect of subjective sleep quality. These authors also found an association between greater variability of sleep duration and high stress levels in adults. Studies on sleep patterns have also highlighted the importance of sleep duration as a mechanism against symptoms of insomnia [47]. Lai [48] showed that sleep quality was associated with less negative effects and greater satisfaction with life due to the link between lack of sleep and loss of mood regulation. Sleep patterns are not only influenced by stress but also by work shifts [49].

An exploratory model of gender differences in depth and hours of sleep is proposed by Kaplan et al. [50], in which efficiency of sleep is one of the variables involved. Equally, the efficiency of better quality sleep (calculated from its duration and time spent in bed) has been linked to greater management of stress by nurses [51]. However, at the present time, there are few studies on how sleep components affect emotion management in multiple mediation models for the effects of sleep duration on stress management and mood.

### 1.2. Research Objectives

This study was intended to analyze the relationships between the components of sleep and emotional intelligence as well as the effects of sleep duration on stress management and mood. That is to determine whether sleep quality mediates in the relationship between sleep duration and the dimensions of emotional intelligence, mood, and stress management in nurses in Andalusia (Spain).

Therefore, on the basis of prior research, we expected to find a significant association between emotional intelligence factors and sleep quality components, the nurses who have less trouble with sleep efficiency to show better stress management and mood, the nurses with better-quality sleep duration to show good mood and stress management; that nurses’ sleep quality depends on the patterns of sleep duration, that is, the number of hours they sleep, and that the efficiency mediates between sleep duration and stress management and mood.

## 2. Materials and Methods

### 2.1. Participants

The original sample was made up of 1094 nurses in Andalusia (Spain). After follow-up and control of the completed questionnaires, those on which incongruent or random answers were detected were discarded, as were any that were incomplete. Therefore, the final study sample was 1073 nursing professionals with a mean age of 32.32 (SD = 6.62), in a range of 22–57 years. Of the whole sample, 14.7% (*n* = 158) were men and 85.3% (*n* = 915) women, with mean ages of 32.79 (SD = 6.27) and 32.24 (SD = 6.68), respectively. By quality of sleep, evaluated by the total *Pittsburgh Sleep Quality Index* (PSQI, [3]; Spanish version by Macías and Royuela [52]) score after previous classification of the variable, participant distribution was 60% (*n* = 644) with sleeping problems and the remaining 40% (*n* = 429) with no sleeping problems.

### 2.2. Instruments

The PSQI questionnaire, which was developed to measure sleep quality, discriminates between good and poor sleepers. It consists of 24 items, five for evaluation by a roommate or bed partner, which are not included in the subject’s self-evaluation score. The 19 self-reported items focus on sleep components, such as sleep latency and duration, frequency, and severity of sleep problems. It is answered with a Likert scale that ranges from 0 to 3. For its correction, a sleep profile is obtained in each of the dimensions (subjective quality, latency, duration, habitual sleep efficiency, disturbances, use of sleep medication, and repercussion on daytime activity), each answer ranging between 0 (no difficulty) and 3 (severe difficulty). The total score of the PSQI was taken for sleep quality, in a range of 0–21 points, where 0 points = No sleep problems and 21 points shows the existence of severe problems in all areas or dimensions evaluated by the instrument. Royuela and Macías [53] reported reliability indices for the instrument of 0.81 with a clinical population and 0.67 in a student sample. In our case, a Cronbach’s Alpha of 0.77 was obtained.

The *Brief Emotional Intelligence Inventory for Senior Citizens* (EQ-i-20M) [54], validated and scaled by the authors for an adult Spanish population, was adapted to adults from the *Emotional Intelligence Inventory: Young Version* (EQ-i-YV) by Bar-On and Parker [55]. It consists of 20 items with four answer choices rated on a Likert-type scale. It is structured in five factors: Intrapersonal (e.g., “I can speak easily about my feelings”), Interpersonal (e.g., “I understand how other people feel”), Stress Management (e.g., “I find it difficult to control my anger”), Adaptability (e.g., “I can solve problems in different ways”), and Mood (e.g., “I am happy with the type of person I am”). The Cronbach’s Alpha for this study was 0.89 for the all the items, and for each of the subscales: 0.90 on Intrapersonal, 0.74 on Interpersonal, 0.82 on Stress Management, 0.81 on Adaptability, and 0.86 on Mood.

### 2.3. Procedure

Before collecting data, compliance with information standards and confidentiality and ethics in data processing were guaranteed to the participants. All subjects gave their informed consent for inclusion before they participated in the study. The study was conducted in accordance with the Declaration of Helsinki and was approved by the Bioethics Committee at the University of Almería. The questionnaires were administered on a Web platform which enabled participants to fill them in online. For control of random or incongruent answers, a series of control questions were included, and any such cases were discarded from the study sample.

### 2.4. Data Analyses

First, to check the relationships between variables, bivariate correlations were calculated. Then, descriptive data on sleep patterns were presented by duration as well as the mean emotional intelligence scores, which have a direct relationship with this parameter.

To estimate the mediation model, the SPSS macro [56] was used in this study for multiple mediation. This resource enables computation of different regression models, finding information on the indirect effects, thereby avoiding the limitations of the classic method by Baron and Kenny [57]. For this, bootstrapping (5000 bootstraps), which enables the determination of any multiple mediation effect of the mediating variables at a 95% confidence interval, was applied. In this study, a multiple mediation analysis with two mediators was performed.

## 3. Results

### 3.1. Sleep and Emotional Intelligence Components: Descriptive and Correlational Analyses

Table 1 shows the correlations between partial sleep quality components and the dimensions of emotional intelligence. For interpretation of the relationships, each of the components has a score of 0 to 3 points, where 0 means there is no problem and 3 shows severe problems, in each case [3,52]. Therefore, when sleep is typified in terms of problems, a negative relationship with another variable would imply, for example, a lower score on the sleep component (less trouble or better quality in that respect), and a higher score on the emotional variable it is associated with.

First, a negative correlation was observed between the Intrapersonal factor and Subjective sleep quality. The Interpersonal factor, on the other hand, had no significant association with any of the sleep quality components. Stress management negatively correlated with all the sleep quality components (subjective sleep quality, sleep latency, sleep duration, sleep disturbances, use of sleeping medication, and daytime dysfunction), except habitual sleep efficiency, where the correlation was positive. In the adaptability factor, the correlations found with sleep disturbances and daytime dysfunction were both negative.

Finally, mood was negatively correlated with all the sleep quality components (subjective sleep quality, sleep latency, sleep duration, sleep disturbances, use of sleeping medication, and daytime dysfunction), except habitual sleep efficiency (*r* = −0.1, *p* = 0.68).

**Figure 1:**
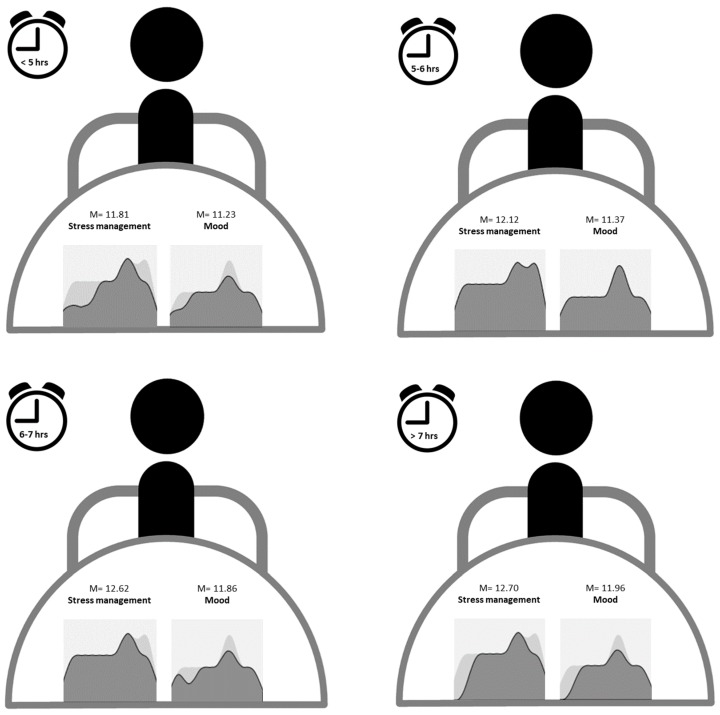
Sleep patterns by duration and mean scores in stress management and mood.

On the basis of the data found in the correlational analyses, stress management and mood were identified as the variables related with sleep duration. Sample distribution by sleep patterns classified by sleep duration showed that 50.3% (*n* = 540) of the participants slept an average of six–seven hours a day, 24.3% (*n* = 261) five–six hours, 21.1% (*n* = 226) over seven hours, and 4.3% (*n* = 46) slept less than five hours. Figure 1 shows the mean scores in stress management (*M*_1073_ = 12.48, SD = 2.23) and mood (*M*_1073_ = 11.74, SD = 2.33), found for each of the sleep patterns. A comparative analysis of means was performed, using the Student’s t-test, in order to compare the scores obtained in stress management and mood, between the groups: <5 h and >7 h of sleep. Statistically significant differences were obtained for stress management (*t* = −2.50, *p* < 0.05), but not for mood (*t* = −1.67, *p* = 0.09) which is tendential. In both cases, those who declared a sleep pattern with a duration >7 h, obtained the highest scores.

There were also differences in the sleep duration patterns between the other sleep quality components (i.e., subjective quality of sleep and sleep efficiency).

In the group of professionals who sleep at least five hours a day, 67.4% evaluated the subjective quality of sleep as “quite poor,” and in 85% of this group, habitual sleep efficiency was below 65%.

The groups with a sleep pattern of 5–6 h and 6–7 h long, showed similar sleep quality characteristics, in both cases “quite good,” and sleep efficiency distributed between the choices of >85% and <65%. Finally, the group of nurses who usually slept more than seven hours did so with 85% efficiency and “quite good” subjective sleep quality.

### 3.2. Multiple Mediation Analysis for the Effects of Sleep Duration on Stress Management and Mood

The mediation analysis was performed based on the following hypotheses:

**H1.**  *The duration of sleep has a direct effect on the perceived effectiveness of sleep and subjective quality, so that a short sleep pattern implies a deterioration in both components of sleep quality.*

**H2a.**  *Difficulties in the perceived effectiveness of sleep has a direct negative effect on Stress Management.*

**H2b.**  *Difficulties in the subjective quality of sleep has a direct negative effect on Stress Management.*

**H2c.**  *The components of sleep quality (efficiency and subjective quality) mediate the relationship between sleep duration and stress management, so that having a short sleep pattern implies a deterioration of sleep quality, which has a negative impact on stress management.*

**H3a.**  *Difficulties in the perceived effectiveness of sleep has a direct negative effect on mood.*

**H3b.**  *Difficulties in the subjective quality of sleep has a direct negative effect on mood.*

**H3c.**  *The components of sleep quality (efficiency and subjective quality) mediate the relationship between sleep duration and mood, so that having a short sleep pattern implies a deterioration of sleep quality, which has a negative impact on mood.*

**Figure 2:**
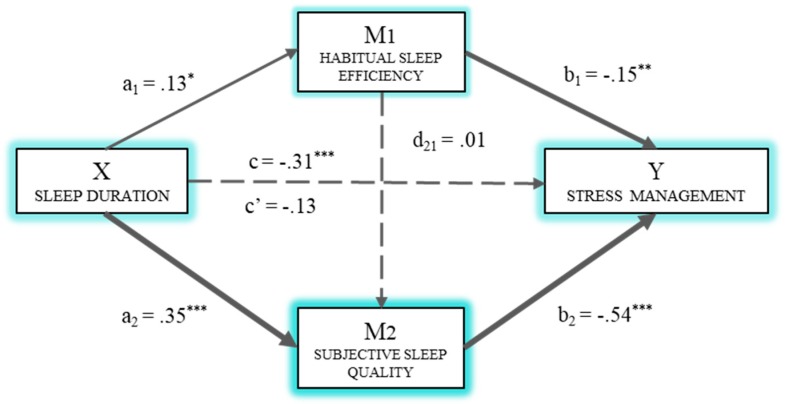
Multiple mediation model of sleep efficiency and subjective quality on the relationship between sleep duration and stress management. * *p* < 0.05; ** *p* < 0.01; *** *p* < 0.001.

To compute the model, the PSQI dimension on sleep duration was taken as the independent variable. The dependent variable included in the first model (Figure 2) was stress management, and as mediators, habitual sleep efficiency (M_1_) and subjective sleep quality (M_2_). Thus, a multiple mediation model was computed with two mediator variables (M_1_: habitual sleep efficiency (HSE) and M_2_: subjective sleep quality (SSQ)). Figure 2 shows the model, including the direct, indirect, and total effects. First, a statistically significant effect (*B*_SD_ = 0.13, *p* < 0.05) of sleep duration (X) on habitual efficiency (M_1_) is observed. The second regression analysis takes Mediator 2 as the result variable and includes sleep duration (X) and habitual efficiency (M_1_) in the equation. Sleep duration had a significant effect (*B*_SD_ = 0.35, *p* < 0.001) on subjective sleep quality (M_2_), but not habitual efficiency (*B*_HSE_ = 0.01, *p* = 0.217). In the third regression analysis, taking stress management (Y) as the result variable, the effect of the predictor variable and the two mediators was estimated. In this case, there were significant effects of both mediators (*B*_HSE_ = 0.15, *p* < 0.01; *B*_SSQ_ = −0.54, *p* < 0.001) on the dependent variable, but there was no significant direct effect (c’) of sleep duration (*B*_SD_ = −0.13, *p* = 0.162). The total effect (c) of sleep quality on stress management was significant (*B*_SD_ = −0.31, *p* < 0.001).

The indirect effects were also analyzed using bootstrapping, which found data supporting a significant level for Path 1 (Ind_1_: X→M_1_→Y; *B* = 0.02, *SE* = 0.01, 95% CI (0.004, 0.048)) and Path 2 (Ind_2_: X→M_2_→Y; *B* = −0.19, *SE* = 0.05, 95% CI (−0.298, −0.101)). Thus, sleep duration had a greater effect on stress management through subjective sleep quality (M2) than through habitual sleep efficiency.

**Figure 3:**
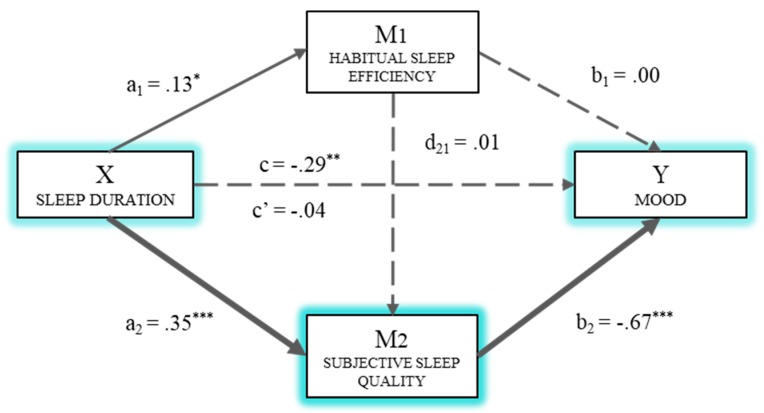
Multiple mediation model of sleep efficiency and subjective sleep quality on the relationship between sleep duration and mood. * *p* < 0.05; ** *p* < 0.01; *** *p* < 0.001.

Figure 3 shows the multiple mediation model for mood as the dependent variable. The effect of the independent variable and the two mediators was estimated based on the third regression analysis. In this case, a significant effect of subjective sleep quality (*B*_SSQ_ = −0.67, *p* < 0.001) on mood was observed, but not of Mediator 1 (*B*_HSE_ = 0.00, *p* = 0.985). Similarly, the direct effect (c’) of duration on the dependent variable was not significant (*B*_SD_ = −0.04, *p* = 0.646). The total effect (c) of sleep duration on mood was significant (*B*_SD_ = −0.29, *p* < 0.01).

Finally, the analysis of indirect effects by bootstrapping extracted data supporting a significant level for Path 3 (Ind_3_: X→M_2_→Y; *B* = −0.24, SE = 0.05, 95% CI (−0.351, −0.143)). Thus, sleep duration has a greater effect on mood through subjective sleep quality (M_2_) than through the two mediators operating in series.

## 4. Discussion

The objective of this study was to examine the relationships between sleep components and emotional intelligence by analyzing the effects of sleep duration on stress management and mood. In addition, we tested whether this relationship was mediated by sleep quality. Thus, we found that on a relational level, the components of sleep quality negatively correlated with stress management and mood, just as occurred in the results found by Lallukka et al. [27] and Sin et al. [28], where sleep duration was negatively related to both variables in adults. In addition, poor sleep quality led to a decreased capacity for regulating emotions and acted as a risk factor causing mood disorders and deficient stress management in healthcare personnel [26]. This corroborates the existence of a significant association between emotional intelligence factors and sleep quality components.

On the other hand, with respect to the quality of sleep of the nurses according to the patterns of sleep duration, it was first discovered that the highest percentage of the sample slept six to seven hours a day [47]. The highest average scores in stress management and mood were those of those nursing professionals who slept 7 h or more. However, there was not much difference in the average score of both emotional intelligence factors between workers who had a sleep duration of between 6 and 7 h and those who slept more. These results affected according to studies that found a link between duration and stress management [46] and lack of sleep and loss of mood regulation [48].

On the basis of the results of the mediation analysis, most of the hypotheses formulated were confirmed. Thus, according to the first hypothesis (H1), a short sleep pattern involved the deterioration of subjective quality and the perceived effectiveness of sleep. This result is in line with the poor quality of sleep reported among people suffering from insomnia [17]. In turn, and according to previous studies [44,45], the alteration of the components of sleep quality had a negative effect on the components of emotional intelligence stress management and mood. Thus, the mediation hypotheses raised in the second place (H2a, H2b, and H2c) were confirmed to find that difficulties in the perceived effectiveness and subjective quality of sleep had a direct effect on stress management and that they mediated the relationship between the duration of sleep and this component of emotional intelligence. However, the strongest mediation effect on stress management was perceived sleep quality, unlike other studies such as that of Kaplan et al. [50], where sleep efficiency is one of the variables of greatest involvement in stress management. Finally, regarding the third group of hypotheses proposed, only H3b could be confirmed, given that in the model of mediation of the quality of sleep on the relationship between its duration and mood, only the subjective quality of sleep had a direct and mediating effect on the component of emotional intelligence.

Therefore, the results found in this study show how sleep duration has an effect on stress management and mood, mainly through the subjective quality of sleep, but also partly because of its effectiveness. That is, health professionals handle stress better and have a more optimistic mood, not so much because of sleep efficiency but because of its quality.

Regarding the limitations of this study, it should be mentioned that it was cross-sectional, which impedes establishing any cause–effect relationships between the variables, so a longitudinal study would still be necessary. The composition of the sample is another of the limitations, since most of the subjects were women, it is therefore hard to generalize the results, although it is also representative of the nursing population, in which the majority are women. In addition, the evaluation of the variables included in the study, carried out through self-completed instruments, may have generated biases such as the effect of variation of the common method in the relationship between sleep quality and mood. Therefore, nursing professionals with sleep disorders and who were emotionally depressed could have attributed their bad mood to poor sleep quality. As for the results, some of the significant correlations found between the components of sleep quality and emotional intelligence were very low, giving little indication as to possible causal inferences. Finally, because of the limited number of studies examining sleep quality in healthcare professionals using mediation models for the effects of sleep duration on stress management and mood, there was only a limited possibility to compare our results with other studies.

Future work should perform studies on the relationship of these variables with work shifts, as it is a factor which must be taken into consideration according to the literature [21,22,49], since nursing personnel react differently to stress and mood.

## 5. Conclusions

The results show the effects of sleep duration on stress management and mood in nursing professionals. Emphasizing the importance of subjective sleep quality as a resource to be taken into consideration for professionals managing stressful situations and mood and improving relationships with their patients and each other.

Moreover, this study provides support for stress management and mood associated with subjective sleep quality as fundamental aspects for health and subjective wellbeing of nursing professionals. Similarly, attention to variables related to the emotional regulation of nursing staff, such as sleep quality, can also have an impact on the care provided to the patient. Therefore, the main finding of this study was that subjective sleep quality, in addition to the number hours of sleep or sleep duration, affects stress management and mood in healthcare professionals. Thus, it is indispensable to make healthcare professionals aware of the importance of sleep quality and develop programs which promote healthy sleep habits.

## Figures and Tables

**Table 1:** Correlations between partial components of sleep quality and emotional intelligence factors.

Construct	Dimensions	SSQ	SL	SD	HSE	SDi	USM	DD	INTRA	INTER	SM	ADAP
**PSQI Sleep quality**	SSQ	–										
	SL	0.41 ***	–									
	SD	0.49 ***	0.25 ***	–								
	HSE	0.07 *	0.07 *	0.07 *	–							
	SDi	0.31 ***	0.27 ***	0.23 ***	0.00	–						
	USM	0.23 ***	0.27 ***	0.16 ***	0.01	0.23 ***	–					
	DD	0.29 ***	0.21 ***	0.23 ***	−0.02	0.31 ***	0.19 ***	–				
**EQi-M-20** **<Emotional intelligence**	INTRA	−0.09 **	−0.04	−0.04	0.01	−0.03	0.03	−0.05	–			
	INTER	−0.00	0.00	−0.00	−0.02	0.01	0.00	0.01	0.37 ***	–		
	SM	−0.15 ***	−0.10 ***	−0.11 ***	0.08 **	−0.16 ***	−0.12 ***	−0.19 ***	−0.00	0.06 *	–	
	ADAP	−0.04	−0.02	−0.00	−0.01	−0.06 *	−0.03	−0.10 **	0.40 ***	0.57 ***	0.04	–
	MO	−0.17 ***	−0.11 ***	−0.09 **	−0.01	−0.17 ***	−0.13 ***	−0.23 ***	0.36 ***	0.35 ***	0.19 ***	0.54 ***

SSQ: Subjective sleep quality; SL: Sleep latency; SD: Sleep duration; HSE: Habitual sleep efficiency; SDi: Sleep disturbances; USM: Use of sleeping medication; DD: Daytime dysfunction; INTRA: Intrapersonal; INTER: Interpersonal; SM: Stress management; ADAP: Adaptability; MO: Mood. * *p* < 0.05; ** *p* < 0.01; *** *p* < 0.001.
